# The Association between the Urinary Excretion of Trimethylselenonium and Trimethylsulfonium in Humans

**DOI:** 10.1371/journal.pone.0167013

**Published:** 2016-11-21

**Authors:** Bassam Lajin, Kevin A. Francesconi

**Affiliations:** Institute of Chemistry–Analytical Chemistry, NAWI Graz, University of Graz, Universitaetsplatz 1, 8010, Graz, Austria; Stony Brook University, Graduate Program in Public Health, UNITED STATES

## Abstract

Hydrogen sulfide is a signaling molecule that plays important roles in several physiological processes, and its methylation product trimethylsulfonium (TMS) is a natural constituent of human urine that could serve as a biomarker for hydrogen sulfide. In vitro studies showed that the enzyme indole-ethylamine N-methyltransferase (INMT) is responsible for the production of trimethylsulfonium as well as its selenium analogue trimethylselenonium (TMSe). Marked inter-individual variability in TMSe production is associated with genetic polymorphisms in the *INMT* gene, but it remains unclear whether these polymorphisms affect substrate specificity or general enzymatic activity. Therefore, we explore the association between the TMS and TMSe production phenotypes. Caucasian volunteers were recruited and grouped according to their TMSe status into “TMSe producers” and “TMSe non-producers”, and morning urine samples were collected over 5 consecutive days from each volunteer. A total of 125 urine samples collected from 25 volunteers (13 TMSe producers and 12 TMSe non-producers) were analyzed for total selenium and total sulfur using inductively coupled plasma mass spectrometry (ICPMS), trimethylselenonium using HPLC/ICPMS, and trimethylsulfonium using HPLC/electrospray ionization—triple quadrupole—mass spectrometry (ESI-QQQ-MS). Although there was no correlation between TMS and TMSe urinary levels within the “TMSe producers” group, the “TMSe producers” had urinary levels of TMS 10-fold higher than those of the “TMSe non-producers” *(P* < 0.001). This result indicates that stratification according to TMSe status or genotype is crucial for the correct interpretation of urinary TMS as a possible biomarker for hydrogen sulfide body pools.

## Introduction

There is growing interest in hydrogen sulfide as a gaseous signaling molecule [1]. The volatility of hydrogen sulfide, however, hinders the investigation of hydrogen sulfide in humans and the need arises for a reliable, stable and easy to measure biomarker for hydrogen sulfide that reflects its *in vivo* levels. We have recently detected for the first time the methylation product of hydrogen sulfide, the trimethylsulfonium ion (TMS), in human urine and proposed its potential utility as an indicator for hydrogen sulfide body pools [2].

The synthesis of TMS is mediated by the enzyme indolethylamine N-methyltransferase, also known as thioether S-methyltransferase [3]. This enzyme produces a variety of products including the selenium analogue of TMS, the trimethylselenonium ion (TMSe), and possesses several key physiological and toxicological functions [3,4]. Several European studies have consistently reported considerable inter-individual variability (20–40 fold) in the human metabolism to TMSe, splitting the study populations into so-called “TMSe producers” and “TMSe non-producers” with a ratio of about 1:4 [5–7].

In 2015, a genome wide association study addressed the production of TMSe in humans [8], with the identification of 3 single nucleotide polymorphisms in the indole-ethylamine N-methyltransferase (*INMT*) gene that were shown to be clearly associated with TMSe urinary levels and explained the inter-individual variability in TMSe production commonly observed in the previous studies. The reported polymorphisms were in perfect linkage disequilibrium (R^2^ = 1), and included 2 polymorphisms in the 3′ untranslated region of *INMT* and one coding non-synonymous polymorphism (rs4720015, phenylalanine→cysteine exchange at codon 253/254) that falls outside the active site of the enzyme, but was predicted to affect protein structure based on *in silico* analyses [8]. Therefore, it is not clear whether these polymorphisms affect substrate specificity: do they affect only the selenium compound or do they impart general enzymatic activity and thereby affect the production of all the enzyme products?

Before making any interpretation of the potential of TMS as a biomarker for hydrogen sulfide, it is essential to assess the inter-individual variability in the production of TMS. Therefore, we investigate in the present study the association between the TMSe production phenotype and TMS levels in human urine.

## Materials and Methods

### Study population

Single urine samples from 59 Caucasian volunteers were collected and screened for TMSe (see below). Thirteen volunteers were found to be TMSe producers; they were recruited into the study along with 12 TMSe non-producers randomly selected from the remaining 46 volunteers. Table 1 shows the characteristics of the two study groups. Each volunteer was asked to donate a morning urine sample (first pass of the day) over 5 consecutive days. Urine was collected in Corning^®^ polypropylene 300 ml sample collection containers (Corning, NY, USA) and divided into several 5 ml portions, which were stored at -80°C until analysis. Volunteers gave informed written consent to participate in the study, and all procedures were in accordance with the Declaration of Helsinki. Medical ethics approval for the conduction of the present study was obtained by the ethics commission at the University of Graz (GZ. 39/10/63).

**Table 1:** Characteristics of the study groups. The values represent the mean ± SD, range.

	TMSe producers (n = 13)	TMSe non-producers (n = 12)
Age (years)	36 ± 11, 19–53	43 ± 12, 26–61
Gender	6 females, 7 males	5 females, 7 males
BMI (kg m^-2^)	24.6 ± 2.7, 21.2–30.1	24.2 ± 2.1, 21.4–29.3

### Elemental determination

A 0.5 ml portion of each urine sample was mineralized using microwave-assisted acid digestion with nitric acid as previously described [9]. The digests were analyzed for total selenium and total sulfur by inductively coupled plasma mass spectrometry (ICPMS, Agilent 7900, Agilent Technologies, Waldbronn). For selenium, the mass spectrometric conditions were as previously described [9]. In brief, the ^78^Se isotope was monitored and we operated in the octopole reaction cell mode with hydrogen as the reaction gas and an optional gas of argon containing 1% CO_2_ to provide a four-fold increase in sensitivity. For sulfur, the ^34^S isotope was measured using conditions similar to those used for selenium except that helium, as a collision gas, replaced hydrogen in the octopole cell. The limits of detection for selenium and sulfur, based on 3*SD of a blank (N = 7) for ^78^Se and ^34^S, were 3 ng L^-1^ and 0.3 mg L^-1^, respectively; Urinary concentrations were normalised according to urine specific gravity determined with a Leica TS 400 total solids refractometer (Leica Microsystems, Buffalo, NY, USA) using the following equation: C_normalized_ = C_measured_ * (SG_average_-1)/(SG_sample_-1) [10].

### The determination of TMS and TMSe

Trimethylselenonium was determined by HPLC/ICPMS as previously described [9], with slight modifications. We used a Hamilton PRP-X200 cation-exchange column (4.1 × 250 mm; Hamilton, Reno, NV, USA), with 20 mM ammonium formate adjusted with formic acid to pH 3.0 as the mobile phase. The mobile phase flow rate was set to 1.4 mL min^-1^ and the column temperature was set to 50°C. The injection volume was 20 μL. A plasma optional gas of 1% CO_2_ in argon was used for the ICPMS detector to enhance the response for Se. Trimethylselenonium was synthesized in-house using a previously described method [11]. The limit of detection for TMSe was 0.8 nM (ca 60 ng Se L^-1^), based on 3*SE_y_, standard error of the y-intercept of a calibration curve recorded over the range of 1–25 nM; precision was <10% for the TMSe in the urine samples.

Trimethylsulfonium was determined by HPLC/electrospray ionization-triple quadrupole mass spectrometry (ESI-QQQ), as previously described [2]. A Hamilton PRP-X200 cation-exchange column (2.1 × 150 mm; Hamilton, Reno, NV, USA) was used with a mobile phase containing 10 mM ammonium formate and 5% acetonitrile, adjusted with ammonia to pH 9.0. The column temperature was set to 30°C and the mobile phase flow rate was set to 0.25 mL min^-1^. Trimethylsulfonium iodide was purchased from Sigma-Aldrich (Vienna, Austria). An isotopically labeled internal standard d_6_-trimethylsulfonium iodide was synthesized in-house as previously described [2,12]. A solution of 100 nM of d_6-_TMS was co-injected as the internal standard (1 μL) using the HPLC autosampler. The triple quadrupole mass analyzer was operated in the selected reaction monitoring mode (SRM), employing the conditions previously described [2]. The limit of detection for TMS (injection volume 1 μL) was 0.2 nM (ca 6 ng S L^-1^), based on 3*SE_y_, standard error of the y-intercept of a calibration curve recorded over the range of 1–10 nM; precision was <10% for TMS in the urine samples.

The thawed urine samples were vortexed prior to filtration through 0.2 μm nylon filters then injected onto the HPLC column. Urinary concentrations of TMS and TMSe were normalized according to specific gravity as described above.

## Results and Discussion

Although TMSe is produced by all humans, individuals clearly fall into two very distinct groups. One group, the “TMSe producers” generally excrete about 10–20% of their urinary selenium as TMSe [5–8], whereas for the second group, “TMSe non-producers”, this value is ≤1% [5,6,8]. In the present study, TMSe was detected (LOD = 0.8 nM) in the TMSe non-producers group but the levels always fell below the limit of quantification (LOQ = 2.6 nM). For TMSe producers, the levels in all urine samples exceeded 24 nM. This gives at least a 10-fold gap in the levels between the two groups and the difference between the average levels of TMSe between the two groups was estimated to be at least 30-fold (Table 2). This variation in TMSe urinary levels is in accordance with that previously reported [5–8].

**Table 2:** Total selenium, total sulfur, trimethylselenonium (TMSe), and trimethylsulfonium (TMS) in the study groups.

	TMSe producers (n = 13)			TMSe non-producers (n = 12)			*P* value
	Mean ± SD (range)	Geo. mean (95% CI)	Median	Mean ± SD range	Geo. mean (95% CI)	Median	
Total S (mg L^-1^)	781 ± 98 (604–956)	775 (718–837)	776	853 ± 129 (599–1004)	844 (762–936)	876	0.110
Total Se (μg L^-1^)	30.4 ± 12.3 (20.5–65.2)	28.7 (23.5–35.1)	25.9	23.9 ± 7.3 (15.3–40.3)	22.9 (19.0–27.6)	23.6	0.113
TMS (nM)	296 ± 202 (76.0–691)	224 (154–364)	222	31.5 ± 24.7 (4.2–69.3)	22.1 (12.1–40.4)	22.3	<0.001
TMSe (nM)*	55.4 ± 19.5 (24.3–93.6)	52.1 (41.7–65.2)	52.2	<LOQ	<LOQ	<LOQ	-

*TMSe was detected in “TMSe non-producers” (LOD = 0.8 nM) but always fell below the limit of quantitation (LOQ = 2.6 nM). The *P* values are two-tailed values calculated using the nonparametric Mann-Whitney *U* test. The urinary level in each volunteer was calculated as the mean for the donated 5 morning urine samples.

**Fig 1:**
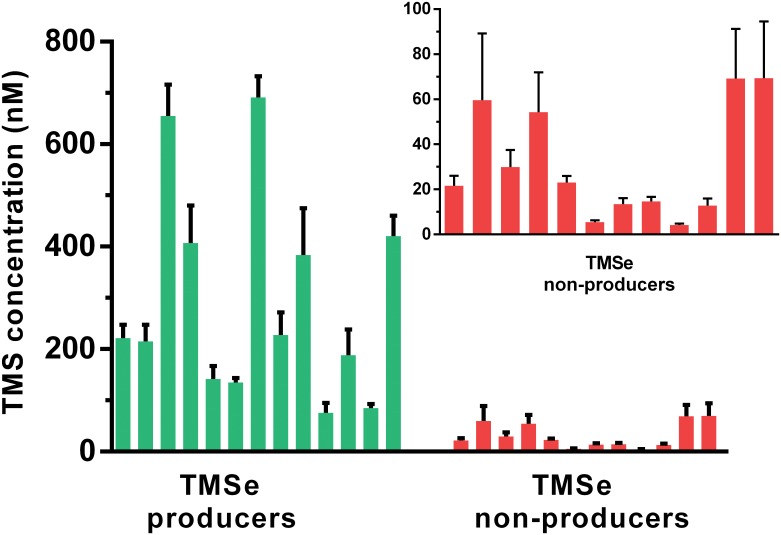
Urinary levels of trimethylsulfonium in the volunteers of the TMSe producers and TMSe non-producers group. Each bar shows the mean of trimethylsulfonium urinary concentration for 5 consecutive days and the standard error of the mean (SEM). The inset shows a zoomed-in portion of the graph for TMS levels in TMSe-nonproducers.

**Fig 2:**
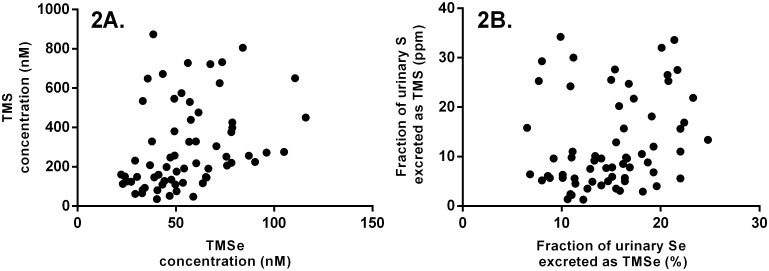
Investigating the correlation between the urinary levels of TMS and TMSe in the TMSe producers group. The TMS levels were expressed as normalized concentrations (A) and fractions of total element excretion (B). Concentrations were normalized according to specific gravity.

The TMS levels were 10-fold higher in the TMSe producers group than those in the TMSe non-producers group (Table 2). This trend was consistent across the volunteers within each group (Fig 1). There was no statistically significant difference in the total urinary sulfur or total urinary selenium between the two groups (Table 2, S1 Fig). No correlation was found between the urinary levels of TMS and TMSe in the TMSe producers group (Fig 2).

Two observations can be made when comparing the patterns in the production of TMSe and TMS. First, the difference in the average TMS levels between the two study groups was three-fold smaller than that observed for TMSe. This result might be at least partly explained by the lower methylation efficiency for DMS compared to DMSe by the INMT enzyme (67 vs. 90 pmol AdoHcy/μg protein/min for DMS and DMSe, respectively) [3]. Additionally, it can also be speculated that the *INMT* polymorphisms might have evolved to affect TMSe to a greater extent than other INMT products. Homology inter-species analyses showed that the alleles associated with high TMSe production are the normal wild type and that they are conserved in the other species [5]. Hence, it seems that up to 75% of the European population evolved to lose the ability to excrete normal levels of TMSe in urine. In the light of the generally low selenium levels in the European soils and the essentiality of this micronutrient in several physiological functions including reproduction, it can be assumed that the evolution of the *INMT* polymorphisms might have been driven to optimize the selenium status.

**Fig 3:**
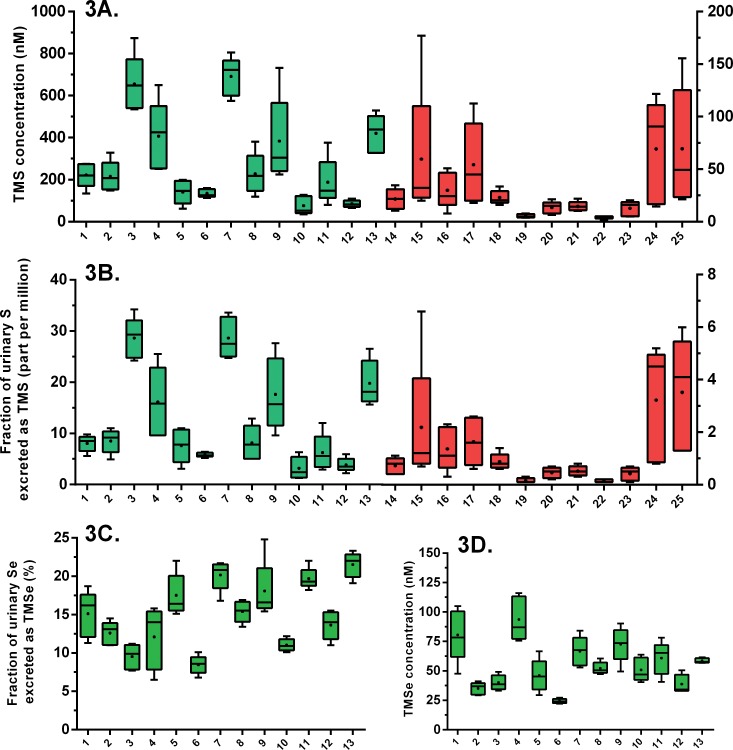
Box and whiskers plot of the concentration of urinary trimethylsulfonium (A), the fraction of total urinary sulfur excreted as trimethylsulfonium (B), fraction of total urinary selenium excreted as trimethylselenonium by TMSe producers (C), and the urinary concentration of trimethylselenonium in TMSe producers (D). Volunteers 1–13 are the TMSe producers (shown in green bars). Volunteers 14–25 are the TMSe non-producers (shown in red bars). TMSe was detected in the TMSe non-producers (LOD = 0.8 nM) but always fell below the limit of quantification (LOQ = 2.6 nM). The graphs show the minimum, maximum, 25% percentile, 75% percentile, arithmetic mean (dot), and median (line) of five consecutive morning urine samples from each volunteer. Concentrations were normalized according to specific gravity.

Second, there was considerable intra-individual and inter-individual variability within each group in the TMS urinary levels (Fig 3A and S1 Table). Total urinary sulfur, however, displayed significantly less pronounced inter-individual and intra-individual variability (S1 Fig). Therefore, normalization according to total urinary sulfur, which is a general indicator of sulfur intake, could not resolve the variability in the TMS levels within each study group (Fig 3B). We calculated the coefficient of variation to assess the inter-individual variability within each of the two study groups for TMSe and TMS. For TMSe, the coefficient of variation was 30% in the TMSe producers group. For TMS, this value was 68% and 78% for the TMSe producers and non-producers, respectively. Apart from INMT activity, several factors can result into inter-individual variability in the urinary TMS/TMSe levels. First, one factor might be the production levels of the precursors DMSe/DMS by the enzyme thiol S-methyltransferase which was found to be polymorphic in humans [13]. This source of variability, however, is expected to affect both TMSe and TMS and the calculated coefficients of variation indicate a clearly higher inter-individual variability in TMS levels within each of the study groups. Therefore, a second source of variability, namely the availability of hydrogen sulfide for methylation, seems to be the dominant factor in the high “within group” inter-individual variability observed for TMS. This may also explain the higher intra-individual variability in TMS than in TMSe (*P* = 0.006). In the TMSe producers group, the intra-individual coefficient of variation in TMSe levels was within the range of 4–30% with a mean of 18%. The intra-individual coefficient of variation in TMS levels in that group was within the range of 13–60% with a mean of 34%.

Tissue hydrogen sulfide is mainly produced from cysteine [14–16] but to avoid its accumulating to toxic levels, hydrogen sulfide is rapidly oxidized to thiosulfate [17,18] thereby maintaining steady-state tissue concentrations in the nanomolar range [17,19]. In our earlier report, we highlighted the possible usefulness of TMS as a biomarker for hydrogen sulfide levels in human body pools [2]. The present study indicates, however, that if such interpretations of urinary TMS levels are to be made (e.g. in case-control studies), then stratification by TMSe status or genotype must be considered.

A recent genome-wide association study revealed the association between TMSe production and three polymorphisms [8]. Since the three polymorphisms are in perfect linkage disequilibrium (R^2^ = 1), it is not clear whether one or more of them are responsible for the variability in TMSe production. Furthermore, the locations of these polymorphisms on the *INMT* gene do not clearly indicate their biochemical effects. Two of the polymorphisms (rs6970396 and rs1061644) are located in the 3′ untranslated region of the gene and were found to cause loss and gain of transcription factor binding sites [8], whereas the coding polymorphism rs4720015 falls outside the active site of the enzyme but was predicted to influence protein structure or function based on *in silico* analysis [8]. Although the TMSe production phenotype was found to directly indicate the INMT genotype (for rs4720015, GG for TMSe non-producers and TG or TT for TMSe producers) [8], it should be noted that owing to the lack of genotype data, the present study does not distinguish between individuals homozygous for the “TMSe producing” variants and heterozygous individuals. However, the frequency of homozygosity for the “TMSe producing” variants in Caucasians is very low (~1% of the population) [20]. Therefore, the group of TMSe producers in the present study is expected to largely consist of heterozygotes. It is noteworthy that the earlier GWAS study which employed a large number of volunteers indicated that the individuals who were homozygous for the TMSe producing variant (n = 10) had only about 1.5 fold higher levels of TMSe than heterozygous individuals [8].

Our results indicate that the polymorphisms in the *INMT* gene affecting TMSe production also influence TMS production, albeit to a lesser extent. Thus it seems likely that these polymorphisms do not produce substrate-specific effects, but rather they influence general enzymatic activity. In that event, the methylation of other substrates in addition to dimethylsulfide and dimethylselenide might be significantly affected. Notably, the effects of these polymorphisms likely extend to other Group 16 elements: tellurium can be methylated by INMT producing trimethyltelluronium from dimethyltelluride [3], and the detoxification product of administered tellurite in rats was found to be trimethyltelluronium [21]. Therefore, the *INMT* polymorphisms might be relevant to occupational exposure to tellurium [22].

Finally, we speculate that the polymorphic enzymatic activity of INMT might also impact on the production of the endogenous psychedelic N, N-dimethyltryptamines through its N-methylation activity [23]. Many studies have investigated the association between the levels of these psychedelic compounds in human bodily fluids, including urine, and psychiatric conditions [24]. Upon examination of the published data, we found no evidence of discontinuous patterns in the reported levels of these compounds in the urine of volunteers [25–27]. This lack of evidence might be a consequence of the complex metabolism of N, N-dimethyltryptamines, which can involve oxidation by monoamine oxidase A into the non-specific metabolite indole-3-acetic acid, excreted in a glucuronide conjugated form, or conversion into their N-oxide derivatives [24]. These alternative fates of the N, N-dimethyltryptamines constitute major confounding sources of variability in their urinary levels, and must be considered in future investigations of the association between the *INMT* polymorphisms and the production of the N, N-dimethyltryptamines.

## Conclusions

Urinary excretion of trimethylsulfonium was found to be associated with that of trimethylselenonium, suggesting that the recently discovered polymorphisms in the *INMT* gene affect the general enzymatic activity. Given the multi-functionality of the INMT enzyme, these polymorphisms are therefore likely to have significant toxicological/physiological consequences. Finally, stratification according to trimethylselenonium status or genotype must be taken into account before considering the possible use of trimethylsulfonium as a biomarker for hydrogen sulfide body pools.

## Supporting Information

S1 Fig Box and whiskers plot of total urinary sulfur (A) and total urinary selenium (B) in the TMSe producers (1–13, green bars) and TMSe non-producers (14–25, red bars). The graph shows the minimum, maximum, 25% percentile, 75% percentile, mean (dot), and median (line). Each volunteer donated 5 morning urine samples. Concentrations were normalized according to specific gravity.
(TIF)

S1 Table The urinary concentrations of TMS in all urine samples analyzed. The intra-individual variability is expressed as the coefficient of variation (CV) for the 5 collected urine samples. The coefficient of variation for the inter-individual variability within the TMSe producers and TMSe non-producers groups were, 68% and 78%, respectively. All concentrations were normalized according to specific gravity.
(DOCX)
