# Supplementary figures and images for: The Association between the Urinary Excretion of Trimethylselenonium and Trimethylsulfonium in Humans
Source: PLoS One. 2016 Nov 21;11(11):e0167013. doi: 10.1371/journal.pone.0167013 (PMC5117766; doi:10.1371/journal.pone.0167013)

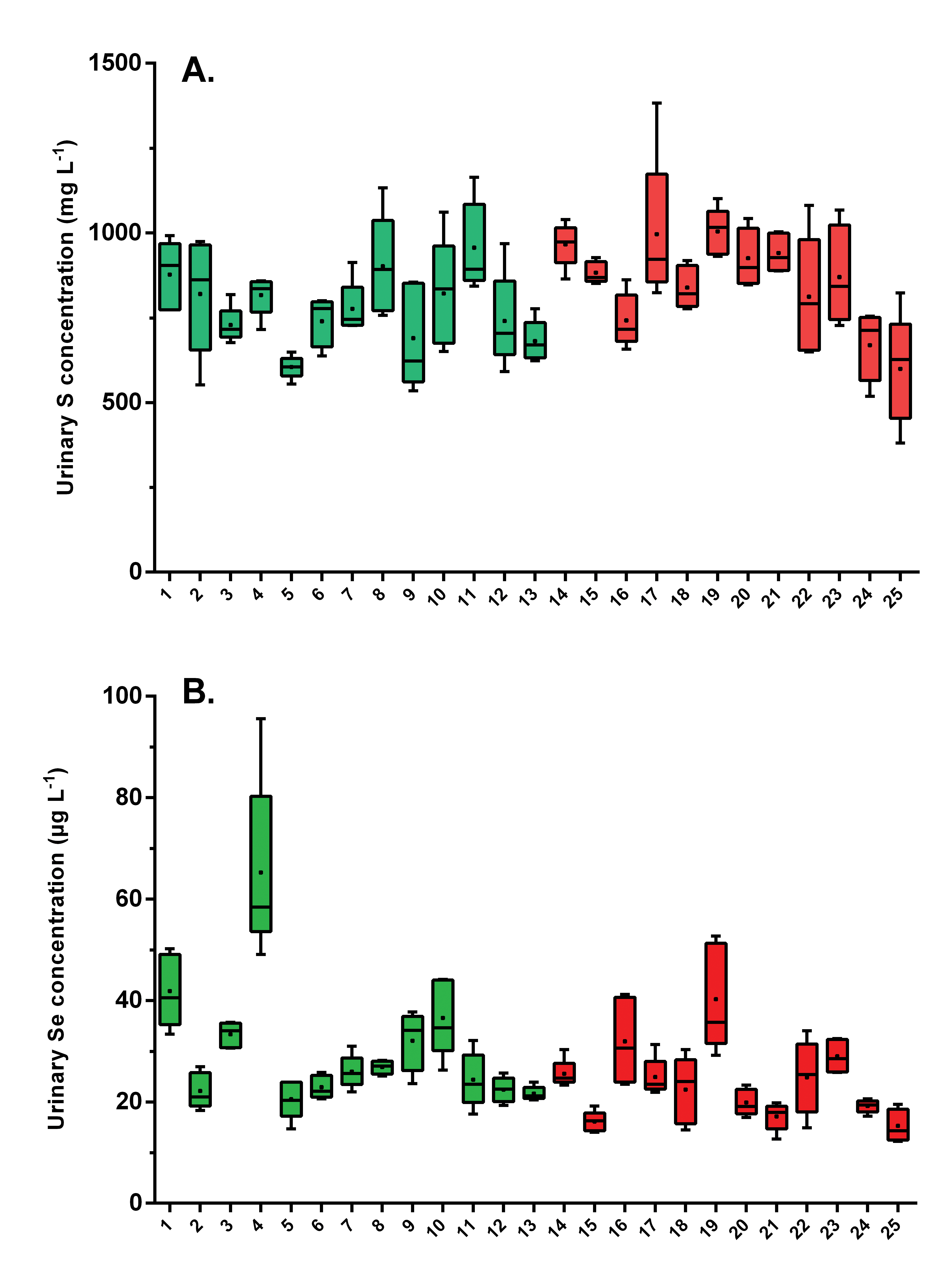

Supplement: S1 Fig — The graph shows the minimum, maximum, 25% percentile, 75% percentile, mean (dot), and median (line). Each volunteer donated 5 morning urine samples. Concentrations were normalized according to specific gravity. (TIF) [file pone.0167013.s001.tif]
