# Supplementary material for: The Association between the Urinary Excretion of Trimethylselenonium and Trimethylsulfonium in Humans
Source: PLoS One. 2016 Nov 21;11(11):e0167013. doi: 10.1371/journal.pone.0167013 (PMC5117766; doi:10.1371/journal.pone.0167013)
Supplement: S1 Table — The intra-individual variability is expressed as the coefficient of variation (CV) for the 5 collected urine samples. The coefficient of variation for the inter-individual variability within the TMSe producers and TMSe non-producers groups were, 68% and 78%, respectively. All concentrations were normalized according to specific gravity. (DOCX) [file pone.0167013.s002.docx]

**S1 Table.** **The urinary concentrations of TMS in all urine samples analyzed.** The intra-individual variability is expressed as the coefficient of variation (CV) for the 5 collected urine samples. The coefficient of variation for the inter-individual variability within the TMSe producers and TMSe non-producers groups were, 68% and 78%, respectively. All concentrations were normalized according to specific gravity.

| **TMSe producers** | | | **TMSe non-producers** | | |
| --- | --- | --- | --- | --- | --- |
| Volunteer# | TMS (nM) | Mean (CV%) | Volunteer# | TMS (nM) | Mean (CV%) |
| 1 | 206 | 222 (26) | 14 | 21.8 | 21.6 (45) |
|  | 276 |  |  | 27.3 |  |
|  | 272 |  |  | 34.6 |  |
|  | 134 |  |  | 14.0 |  |
|  | 221 |  |  | 10.5 |  |
| 2 | 149 | 215 (33) | 15 | 20.1 | 59.6 (111) |
|  | 159 |  |  | 177 |  |
|  | 208 |  |  | 32.3 |  |
|  | 329 |  |  | 43.0 |  |
|  | 231 |  |  | 25.7 |  |
| 3 | 874 | 655 (21) | 16 | 50.9 | 29.9 (57) |
|  | 672 |  |  | 7.8 |  |
|  | 546 |  |  | 24.0 |  |
|  | 535 |  |  | 24.4 |  |
|  | 649 |  |  | 42.2 |  |
| 4 | 451 | 407 (41) | 17 | 17.9 | 54.3 (73) |
|  | 651 |  |  | 74.3 |  |
|  | 256 |  |  | 44.9 |  |
|  | 251 |  |  | 113 |  |
|  | 426 |  |  | 22.0 |  |
| 5 | 190 | 141 (40) | 18 | 24.8 | 23.0 (29) |
|  | 110 |  |  | 20.4 |  |
|  | 62.4 |  |  | 16.0 |  |
|  | 199 |  |  | 20.3 |  |
|  | 146 |  |  | 33.5 |  |
| 6 | 113 | 135 (15) | 19 | 4.7 | 5.5 (31) |
|  | 151 |  |  | 7.7 |  |
|  | 125 |  |  | 6.9 |  |
|  | 161 |  |  | 4.3 |  |
|  | 124 |  |  | 3.9 |  |
| 7 | 575 | 691 (13) | 20 | 9.2 | 13.5 (43) |
|  | 728 |  |  | 6.6 |  |
|  | 722 |  |  | 21.4 |  |
|  | 625 |  |  | 15.1 |  |
|  | 806 |  |  | 15.2 |  |
| 8 | 247 | 228 (43) | 21 | 15.4 | 14.6 (31) |
|  | 176 |  |  | 11.5 |  |
|  | 381 |  |  | 10.2 |  |
|  | 218 |  |  | 21.9 |  |
|  | 119 |  |  | 14.2 |  |
| 9 | 225 | 383 (54) | 22 | 5.3 | 4.2 (32) |
|  | 399 |  |  | 4.0 |  |
|  | 305 |  |  | 4.6 |  |
|  | 257 |  |  | 2.0 |  |
|  | 732 |  |  | 5.2 |  |
| 10 | 116 | 76.0 (54) | 23 | 4.9 | 12.8 (0.56) |
|  | 35.6 |  |  | 5.3 |  |
|  | 47.7 |  |  | 16.1 |  |
|  | 128 |  |  | 17.1 |  |
|  | 52.4 |  |  | 20.4 |  |
| 11 | 145 | 188 (60) | 24 | 122 | 69.2 (71) |
|  | 148 |  |  | 100 |  |
|  | 191 |  |  | 19.2 |  |
|  | 80.3 |  |  | 90.6 |  |
|  | 376 |  |  | 14.4 |  |
| 12 | 110 | 85.1 (20) | 25 | 49.3 | 69.3 (81) |
|  | 82.2 |  |  | 95.1 |  |
|  | 65.1 |  |  | 156 |  |
|  | 75.1 |  |  | 25.6 |  |
|  | 93.0 |  |  | 21.2 |  |
| 13 | 530 | 420 (21) |  |  |  |
|  | 439 |  |  |  |  |
|  | 327 |  |  |  |  |
|  | 329 |  |  |  |  |
|  | 476 |  |  |  |  |
